# Changes in Serum Creatinine Levels and Natural Evolution of Acute Kidney Injury with Conservative Management of Hemodynamically Significant Patent Ductus Arteriosus in Extremely Preterm Infants at 23–26 Weeks of Gestation

**DOI:** 10.3390/jcm9030699

**Published:** 2020-03-04

**Authors:** Eun Seop Seo, Se In Sung, So Yoon Ahn, Yun Sil Chang, Won Soon Park

**Affiliations:** Department of Pediatrics, Samsung Medical Center, Sungkyunkwan University School of Medicine, 06351 Seoul, Korea; eunseop720@gmail.com (E.S.S.); sein.sung@samsung.com (S.I.S.); soyoon.ahn@samsung.com (S.Y.A.); yschang@skku.edu (Y.S.C.)

**Keywords:** acute kidney injury, patent ductus arteriosus, conservative management

## Abstract

Changes in kidney function in extremely preterm infants (EPT) with conservatively managed hemodynamically significant (HS) patent ductus arteriosus (PDA) are not known well. We aimed to present the postnatal course in serum creatinine levels (sCr), prevalence of acute kidney injury (AKI), then relevance between AKI and adverse outcomes in EPT with conservatively managed HS PDA. By review of medical records, we analyzed the postnatal course of sCr and prevalence of stage 3 AKI defined by the modified Kidney Disease Improving Global Outcome (KDIGO) in EPT at gestational age of 23 to 26 weeks with conservatively treated HS PDA. We investigated if the presence and/or prolonged duration of stage 3 AKI elevated the risk of adverse outcomes. The results showed that, neither factor was associated with adverse outcomes. While the average PDA closure date was at postnatal day (P) 41 and 53, sCr peaked at P 10 and 14 and the cumulative prevalence of stage 3 AKI was 57% and 72% in the EPT of 25–26 and 23–24 weeks’ gestation, respectively. The high prevalence of stage 3 AKI without adverse outcomes in EPT with conservatively managed HS PDA suggests that it might reflect renal immaturity rather than pathologic conditions.

## 1. Introduction

Assessing kidney function is crucial for meticulous fluid, electrolyte, and nutritional support, and the adjustment of medication dosage in extremely preterm infants (EPT) [1,2,3,4]. Serum creatinine level (sCr) is a commonly used in evaluating renal function and could also be applied in assessment of glomerular filtration rate (GFR) in neonates and infants [4,5,6,7]. However, the use of sCr for renal function assessment in preterm infants is problematic as their sCr at birth reflects maternal levels [8,9], and sCr is quite variable according to gestational age (GA), birth weight, and chronological age [4,7,10,11]. Limited data are available on how sCr is affected by gestational age and birth weight and how this value changes over time, especially in the peri-viable EPT [5,7,10,12,13,14]. Despite these limitations, all the three current available acute kidney injury (AKI) definitions use change in sCr to classify the stage of AKI in the newborn infants [5,15,16].

AKI in premature infants are known to be related to increased mortality [11,17,18,19,20,21] and morbidities, which includes bronchopulmonary dysplasia (BPD) [2,22,23] and intraventricular hemorrhage (IVH) [24]. However, these associations have not been well reported and elucidated in EPT, although EPT are at high risk for acute AKI because of low GFR resulting from under-developed kidney systems, exhibiting incomplete nephrogenesis and low nephron number [25,26]. Meanwhile, hemodynamically significant (HS) patent ductus arteriosus (PDA) could promote developing AKI by decreasing renal perfusion in the preterm infants in recent studies [16,17,27,28,29]. However, growing evidences support that the conservative management of HS PDA could be safe and feasible without increased mortality and/or morbidities [30,31,32,33]. Furthermore, the risks of developing AKI and the ensuing adverse outcomes with the conservative management of HS PDA have not yet been delineated. Therefore, we conducted this investigation to provide the natural postnatal course of changes in sCr, and the prevalence of AKI in EPT at gestation of 23–26 weeks with HS PDA exclusively managed with a conservative approach [31,32]. We also examined if the presence or persistence of AKI stage 3 adversely affected the risk of adverse events by comparing mortality and morbidities between EPT with and without AKI stage 3.

## 2. Experimental Section

### 2.1. Study Sample

The Samsung Medical Center (SMC) Institutional Review Board approved our investigation and waived the need for consent on October 10, 2019 (No. SMC 2015-10-156). We reviewed medical charts of 97 EPT at gestation of 23–26 weeks admitted to our Neonatal Intensive Care Unit (NICU) from January 2011 to June 2014 presenting with HS PDA, and treated exclusively by a conservative approach [31,32]. We stratified the extremely preterm infants into 23–24 (*n* = 50) and 25–26 (*n* = 47) weeks’ gestation, and analyzed rates of mortality and morbidities, such as necrotizing enterocolitis (NEC), BPD, and intraventricular hemorrhage (IVH) in accordance with the presence/absence of and duration of AKI stage 3 [5,16,18].

### 2.2. AKI

AKI events occurring during the 6-week postnatal period were detected by the neonatal modified KIDGO sCr criteria [5,16,18] (Table 1). Measuring a chemistry panel including sCr q 1–3 days is usual at our NICU if the infant’s condition is critical during the first few weeks of life, and increasing the interval up to q 1–2 weeks, if the infant’s condition has become stabilized. Although we did not adopt urine amount criteria to classify stage, we calculated urine output from flow sheets, and reported the incidence of oliguria (<0.5 mL/kg/day) at each stage of AKI.

### 2.3. HS PDA

We defined HS PDA as more than 2 mm in ductal diameter plus predominant left to right flow on echocardiography initially performed at average postnatal day 7; requiring ventilator support accompanying signs and symptoms consistent with symptomatic PDA, such as hypotension with mean airway pressure below GA; grade ≥ 2 cardiac murmur; pulse pressure widening (>30 mmHg); or need for increased respiratory support [31,32]. We deferred until postnatal day 7 as spontaneous ductal closures could occur even in EPT for the first postnatal week [17,34]. Follow-up echocardiography was conducted regularly at 2–4 weeks intervals until PDA closure. During the study period, 50/54 (93%) and 47/74 (64%) in the EPT of 23–26 weeks of gestation were diagnosed with HS PDA, respectively.

### 2.4. Fluid Therapy

We managed all EPT with HS PDA with non-interventional conservative management without any pharmacologic and/or surgical intervention. We judiciously restricted the fluid intake starting with the first-day mean fluid volume around 67 mL/kg/day, and maintaining mean fluid intake around 107–115 mL/kg/day from days 7 to 28 for the first two months of life [31,32]. We individualized and adjusted the target fluid volume for each EPT q 24 h after assessment of volume status by body weight, serum sodium level, urine output and specific gravity, or cardiomegaly. In this present study, we could obtain judicious fluid restriction in EPT through meticulous NICU care including better room care delivery, minimal handling, and high humidification [35,36].

### 2.5. Data Collection and Definition

We analyzed clinical characteristics, which included sex, birth weight, GA, Apgar score at 1-min and 5-min, mode of delivery, chorioamnionitis, use of inotropic drugs, antenatal steroid use, and oliguria. We determined GA using the last menstrual period of mother and modified Ballard score. We confirmed chorioamnionitis using placental pathology. We reported oliguria when urine amount is less than 0.5 mL/kg for a day.

We analyzed adverse outcomes including ≥ moderate BPD [37], cystic periventricular leukomalacia, IVH (grade ≥ 3) [38], NEC (Bell’s stage ≥ 2b) [39], retinopathy of prematurity (ROP) (stage ≥ 3) [40], and mortality.

To present the time course of sCr and AKI by gestational age, cumulative incidence rates of AKI in EPT at gestational age of 23–24 and 25–26 weeks were evaluated. We measured the adjusted odds ratios (ORs) of mortality and morbidities by the presence and/or persistence (per increase of week) of AKI stage 3 using multivariate regression analyses.

### 2.6. Statistical Analyses

We analyzed the categorical variables by χ^2^ tests and Fisher’s exact test. For continuous variables, we analyzed data through Student’s *t*-tests and Mann–Whitney *U* tests. We also did multivariable analyses by binary logistic regression to measure adjusted ORs and 95% CI of the association between the duration of stage 3 AKI and adverse outcomes including mortality within the entire cohort. We considered a *p* value less than 0.05 as statistically significant. We used SPSS version 21 (SPSS Inc., Chicago, IL, USA) in all data analyses.

## 3. Results

### 3.1. Natural Course of sCr

**Figure 1:**
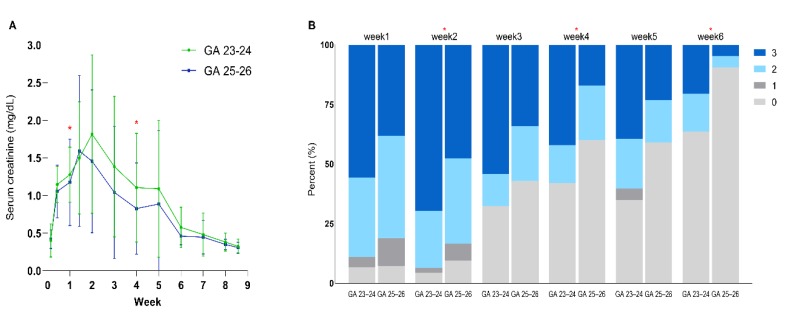
Time course of the mean serum creatinine levels within different gestational groups and prevalence of acute kidney injury (AKI) by stage in a week interval. (**A**) Serum creatinine profile for the 6 weeks of life in accordance to different gestational age groups; (**B**) Prevalence of AKI by stage in a week interval. * *p* < 0.05 in comparison between infants at 23–24 and those at 25–26 weeks of gestation.

For the time course of sCr, the initial increase in sCr peaked at postnatal day (P) 10 and postnatal week 2 in EPT at 25–26 and 23–24 weeks of gestation, respectively (Figure 1A). The peak sCr showed a higher tendency without statistical significance in EPT at gestation of 23–24 weeks than in those at 25–26 weeks. After this, sCr gradually declined until postnatal week 9 in both subgroups and reached sCr at birth at postnatal week 6 and 7 in EPT at 25–26 and 23–24 weeks’ gestation, respectively. In EPT of 25–26 weeks’ gestation without HS PDA, sCr showed a similar time course without statistical significance with the EPT of 25–26 weeks’ gestation with HS PDA (Supplementary Figure S1).

### 3.2. AKI Prevalence

Table 1 demonstrates the cumulative AKI stage within the first six postnatal weeks according to neonatal KDIGO classification stratified by gestational age group. While only 6% and 8% were at AKI stage 0 in EPT at 25–26 and 23–24 weeks of gestation, respectively, the prevalence of AKI stage 3 tended to be higher (72%) in EPT at gestation of 23–24 weeks than in EPT of 25–26 weeks (57%) without statistical significance.

For the time course of the prevalence of AKI by stage in a week interval, postnatal increase in the prevalence of AKI stage 3 peaked at postnatal week 2 in both groups, and afterwards prevalence gradually declined till postnatal week 6 (Figure 1B). The prevalence of AKI stage 3 at postnatal week 2 and 6 in EPT at gestation of 23–24 weeks was higher significantly than that in EPT at gestation of 25–26 weeks.

### 3.3. Clinical Characteristics According to AKI Stage

Demographic and clinical characteristics in each study group in accordance with AKI stages are described in Table 2. In EPT with AKI stage 3, total GA was significantly lower, and male gender in EPT at gestation of 25–26 weeks had higher GA than in EPT with AKI 0–2. While total oliguria and oliguria in EPT at 25–26 weeks of gestation with AKI stage 3 were higher compared with those with AKI stage 0–2 significantly, no differences were observed in other clinical variables between AKI stage 3 and stage 0–2 groups.

### 3.4. Adverse Outcomes According to AKI Stage

While sepsis in EPT at 25–26 weeks of gestation with AKI stage 3 was slightly higher than infants with AKI stage 0–2, no significant differences were found in other adverse outcomes, including mortality and BPD, between the AKI stage 3 and stage 0–2 groups (Table 3).

### 3.5. Adjusted ORs for Risk of Adverse Outcomes by AKI Stage 3

The adjusted ORs for the risk of unfavorable outcomes were not increased in AKI stage 3 in multivariate analyses (Table 4). In addition, the adjusted ORs for outcomes were not elevated by prolonged duration (per week) of AKI stage 3 (Table 5).

## 4. Discussion

This present study is the first human study demonstrating the natural postnatal evolution of sCr and the prevalence of AKI in the peri-viable EPT at gestation of 23–26 weeks with HS PDA who received exclusive conservative management. In this present study, while sCr at birth was about the same between the study groups, representing maternal levels [8,9], the peak sCr and the peak prevalence of AKI stage 3 during the first two postnatal weeks were higher in EPT at gestation of 23–24 weeks than in EPT of 25–26 weeks. These findings suggest that very low initial GFR and tubular immaturity, and its slow improvement, might be inversely related to GA in these peri-viable EPT [7,11,14,27,41]. Furthermore, as HS PDA closed averagely at postnatal day 41 and 53 in EPT of 25–26 and 23–24 weeks’ gestation, respectively [31,32], our data suggests that renal immaturity inversely related to GA, rather than HS PDA induced renal hypo-perfusion [17,27,28,29], are primarily responsible for the initial postnatal rise in sCr and the peak prevalence of AKI stage 3 during the first two postnatal weeks [42].

sCr after peak declined rapidly, and reached a birth sCr at postnatal week 6 and 7, compatible with corrected GA of 31–32 weeks in EPT at 25–26 and 23–24 weeks of gestation, respectively, and after then, sCr approached a stable plateau in both GA subgroups, indicating a steady state between endogenous Cr production and excretion [13,27,41]. In the preterm infant, the GFR is lower until the full nephrogenesis is finished by 34–35 weeks of gestation [43,44,45,46]. Overall, our data suggest that despite its inverse relationship with GA, the postnatal renal maturation and the ensuing logarithmic increase in GFR are accelerated by 2–3 weeks in these peri-viable EPT [47].

Although AKI in premature infants has been known to be related to raised mortality [11,17,18,19,20,21] and morbidity rates, including BPD [2,22,23] and IVH [24], evidence supporting their direct causal relationships are lacking. In contrast, while sepsis rate was more elevated in AKI stage 3 than in stage 0–2, only in EPT at gestation of 25–26 but not of 23–24 weeks, neither the presence nor the prolonged duration of AKI stage 3 was associated with elevated mortality or any morbidities rates including BPD and IVH. The reasons for our results, which are contradictory to other studies showing increased mortality and/or morbidities [2,11,17,18,19,20,21,22,23,24], are difficult to explain. Few data are available for the peri-viable EPT of 23–24 week’s gestation, and actively treated with pharmacologic agents for HS PDA could be cofounders in other studies. A further controlled study with a homogeneous patient population and same clinical management policy might be necessary to clarify these contradictory findings.

Fluid therapy and drug dosing in premature infants need to be adjusted according to renal function, i.e., GFR [3,4,45,46]. Considering our results, which showed greater and delayed peak of sCr and very high prevalence of AKI stage 3 at the first two postnatal weeks in these peri-viable EPT with HS PDA, judicious fluid restriction might be prerequisite for the success of non-interventional treatment for HS PDA [2,48,49]. In our prior studies [31,32], fluid volume of 67 mL/kg/day at day of birth, and raising up to ≤115 mL/kg/day for the first month was accomplished without restricting caloric support or elevating the risk of renal dysfunctions and electrolyte imbalance. Acute fluid overload in the newborn infants was associated with adverse outcomes, including mortality [50] and morbidities [2,48]. Furthermore, the extent of volume overload in critical adult patients also correlated with worse clinical course [51,52,53]. In contrast, fluid restriction was associated with reduced mortality [54] and morbidities, such as PDA and NEC [55]. Overall, these findings suggest that judicious fluid restriction might be essential for the success of the non-interventional conservative treatment of HS PDA in EPT [45].

Heterogeneous time intervals and variable number of follow-up sCr measurements for review could be limitations of this retrospective uncontrolled observational single center study. The absence of long-term outcome assessments including growth and neurodevelopment might be another limitation of this study. However, a relatively large sample size (*n* = 50) of the peri-viable EPT at gestation of 23–24 weeks with HS PDA exclusively managed with a conservative treatment, as well as less variation in clinical management policies, might be a strength of this single-center study.

## 5. Conclusions

In conclusion, the study findings suggest that AKI observed in EPT with conservatively managed HS PDA is not a pathological entity and might reflect a physiological postnatal developmental process of the immature renal system.

## Figures and Tables

**Table 1:** The maximum AKI stage within a first month after birth according to neonatal acute kidney injury KDIGO classification.

Stage	Serum Creatinine	GA 23–24 Weeks *n* = 50	GA 25–26 Weeks *n* = 47	Total *n* = 97	Total with Oliguria (<0.5 mL/kg/day) *n* = 97
0	No change in SCr or rise < 0.3 mg/DL	4 (8%)	3 (6%)	7(7%)	1(1%)
1	SCr rise ≥ 0.3 mg/dL within 48 h or SCr rise ≥ 1.5–1.9 × reference SCr ^a^ within 7 days	2 (4%)	5 (11%)	7(7%)	1(1%)
2	SCr rise ≥ 2.0–2.9 × reference SCr ^a^	7 (14%)	11 (23%)	18(19%)	1(1%)
3	SCr rise ≥ 3 × reference SCr ^a^ or SCr ≥ 2.5 mg/dL ^b^ or receipt of dialysis	36 (72%)	27 (57%)	63(66%)	18(19%)

^a^ Reference SCr will be considered as the lowest prior SCr value. ^b^ SCr value of 2.5 mg/dL corresponds to GFR less than 10 mL/min/1.73 m^2^. AKI, acute kidney injury; SCr, serum creatinine; KDIGO, Kidney Disease Improving Global Outcomes.

**Table 2:** Demographics and clinical characteristics of EPT in period II: Stage 0–2 AKI vs. stage 3 AKI.

Clinical Characteristics	Total (*n* = 97)					
	GA 23–24 Weeks (*n* = 50)		GA 25–26 Weeks (*n* = 47)		Total (*n* = 97)	
	AKI 0–2 (*n* = 13)	AKI 3 (*n* = 36)	AKI 0–2 (*n* = 18)	AKI 3 (*n* = 27)	AKI 0–2 (*n* = 31)	AKI 3 (*n* = 63)
Gestational age (weeks)	23.9 ± 0.4	23.6 ± 0.5	25.6 ± 0.5 ^†^	25.3 ± 0.4 ^†^	24.8 ± 1.0	24.3 ± 1.0 *
Birth weight, mean (SD), g	684 ± 90	636 ± 79	743 ± 145	829 ± 140 ^†^	718 ± 127	719 ± 145
Male, *n* (%)	6(46)	19(53)	7(39)	19(70) *	13(42)	38(60)
Apgar score at 1-min	3.9 ± 0.7	4.2 ± 1.3	4.4 ± 1.8	4.7 ± 1.4	4.2 ± 1.4	4.4 ± 1.4
Apgar score at 5-min	6.9 ± 1.1	6.5 ± 1.4	6.8 ± 1.4	6.9 ± 1.5	6.8 ± 1.3	6.7 ± 1.5
Cesarean delivery, *n* (%)	8 (62)	2 6(72)	16 (89)	24 (89)	24 (78)	50 (79)
Hypertension in pregnancy, *n* (%)	0	0	1 (6)	0	1 (3)	0
Chorioamnionitis, *n* (%)	6 (46)	25 (69)	9 (50)	15 (56)	15 (48)	40 (62)
Use of inotropic drugs, *n* (%)	3 (23)	8 (22)	4 (22)	1 (4)	7 (44)	9 (56)
Antenatal steroid use, *n* (%)	12 (92)	27 (75)	13 (72)	24 (89)	25 (81)	51 (81)
Oliguria, *n* (%)	1 (8)	9 (25)	1 (6)	9 (33) *	2 (7)	18 (29) *

* *p* < 0.05 compared with Stage 0–2 AKI. † *p* < 0.05 compared with infants at 23–24 weeks of gestation.

**Table 3:** Adverse outcomes of infants in period II: stage 0–2 AKI vs. stage 3 AKI.

Adverse Outcomes	Total (*n* = 97)					
	GA 23–24 Weeks (*n* = 50)		GA 25–26 Weeks (*n* = 47)		Total (*n* = 97)	
	AKI 0–2 (*n* = 13)	AKI 3 (*n* = 36)	AKI 0–2 (*n* = 18)	AKI 3 (*n* = 27)	AKI 0–2 (*n* = 31)	AKI 3 (*n* = 63)
Mortality, *n* (%)	1 (8)	7 (19)	1 (6)	1 (4)	2 (7)	8 (13)
Length of stay	111 ± 14	120 ± 64	130 ± 68	110 ± 59	122 ± 53	116 ± 62
NEC (Stage ≥ 2b), *n* (%)	0	5 (14)	2 (11)	3 (11)	2 (7)	8 (13)
ROP (requiring laser operation), *n* (%)	3 (23)	10 (28)	5 (28)	7 (26)	8 (26)	17 (27)
Blood culture-proven sepsis, *n* (%)	3 (23)	13 (36)	1 (6)	10 (37) *	4 (13)	23 (37) *
Cystic PVL, *n* (%)	3 (23)	7 (20)	3 (17)	2 (7)	6 (19)	9 (15)
IVH (Grade ≥ 3), *n* (%)	1 (8)	8 (22)	1 (6)	2 (7)	2 (7)	10 (16)
BPD (≥moderate BPD), *n* (%)	5 (39)	15 (47)	6 (33)	8 (30)	11 (36)	23 (39)
Survival without BPD, *n* (%)	1 (0)	0	1 (6)	3 (11)	2 (7)	3 (5)

* *p* < 0.05 compared with Stage 0–2 AKI.

**Table 4:** Adjusted ORs * for risk of adverse outcomes by presence of Stage 3 AKI.

Outcomes	Adjusted OR (95% CI)	*p* Value
Mortality	0.965 (0.140–6.661)	0.971
BPD (more than moderate BPD)	1.441 (0.507–4.095)	0.493
Survival without BPD	0.314 (0.018–5.559)	0.430
IVH (Grade ≥ 3), *n* (%)	1.923 (0.360–10.269)	0.444
Cystic PVL	0.460 (0.116–1.819)	0.268
ROP (requiring laser operation), *n* (%)	1.538 (0.480–4.926)	0.469
NEC (Stage ≥ 2b), *n* (%)	3.610 (0.439–29.654)	0.232
Blood culture-proven sepsis	3.556 (0.965–13.101)	0.057

OR, odds ratio; AKI, acute kidney injury; CI, confidence interval; BPD, bronchopulmonary dysplasia; IVH, intraventricular hemorrhage; PVL, periventricular. leukomalacia; ROP, retinopathy of prematurity; NEC, necrotizing enterocolitis. * adjusted for birth weight, gestational age, small for gestational age, antenatal steroid use, 1-min and 5-min Apgar scores, hypertension in pregnancy, chorioamnionitis.

**Table 5:** Adjusted ORs * for risk of adverse outcomes by duration (per week) of stage 3 AKI.

Outcomes	Adjusted OR (95% CI)	*p* Value
Mortality	1.040 (0.602–1.797)	0.887
BPD (more than moderate BPD)	1.043 (0.745–1.459)	0.808
Survival without BPD	0.332 (0.083–1.329)	0.119
IVH (Grade ≥ 3), n (%)	1.164 (0.074–1.823)	0.508
Cystic PVL	0.709 (0.437–1.150)	0.163
ROP (requiring laser operation), n (%)	1.000 (0.693–1.441)	0.998
NEC (Stage ≥ 2b), n (%)	1.325 (0.748–2.346)	0.335
Blood culture-proven sepsis	1.170 (0.820–1.665)	0.382

OR, odds ratio; AKI, acute kidney injury; CI, confidence interval; BPD, bronchopulmonary dysplasia; IVH, intraventricular hemorrhage; PVL, periventricular. leukomalacia; ROP, retinopathy of prematurity; NEC, necrotizing enterocolitis. * adjusted for birth weight, gestational age, small for gestational age, antenatal steroid use, 1-min and 5-min Apgar scores, hypertension in pregnancy, chorioamnionitis.

## Supplementary Materials

The following are available online at https://www.mdpi.com/2077-0383/9/3/699/s1, Figure S1: Time course of the mean serum creatinine levels between infants with HS (hemodynamically significant) PDA (patent ductus arteriosus) and those with Non HS PDA for 60 days of life.
