# Supplementary figures and images for: Changes in Serum Creatinine Levels and Natural Evolution of Acute Kidney Injury with Conservative Management of Hemodynamically Significant Patent Ductus Arteriosus in Extremely Preterm Infants at 23–26 Weeks of Gestation
Source: J Clin Med. 2020 Mar 4;9(3):699. doi: 10.3390/jcm9030699 (PMC7141372; doi:10.3390/jcm9030699)

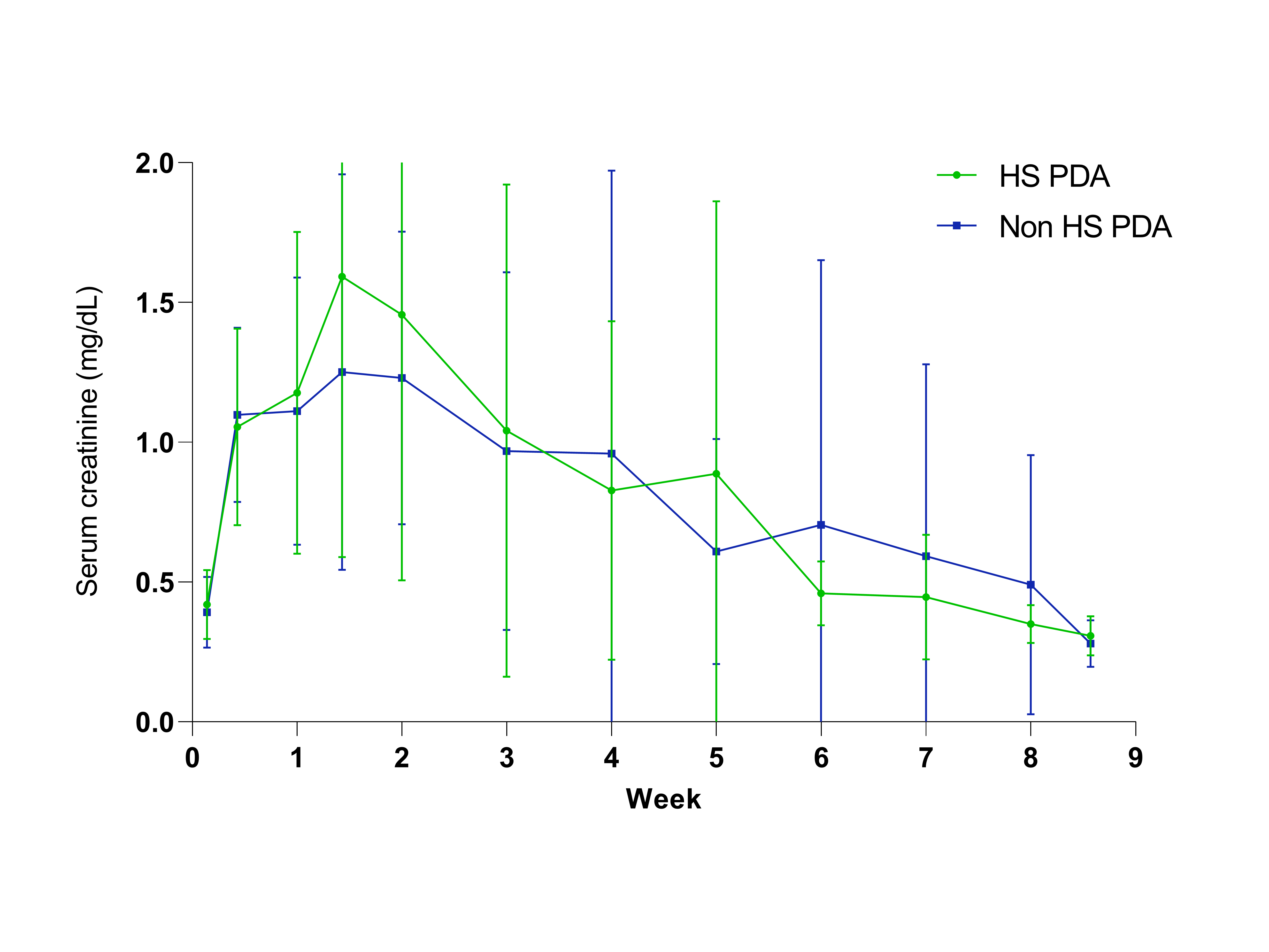

Supplement: Supplementary file 1 [file jcm-09-00699-s001.zip › jcm-728023-supplementary.tif]
